# Antigen-specific CD4^+^ T cells promote monocyte recruitment and differentiation into glycolytic lung macrophages to control *Mycobacterium tuberculosis*

**DOI:** 10.1371/journal.ppat.1013208

**Published:** 2025-06-09

**Authors:** Samuel H. Becker, Christine E. Ronayne, Tyler D. Bold, Marc K. Jenkins

**Affiliations:** 1 Center for Immunology, Department of Microbiology and Immunology, University of Minnesota Twin Cities School of Medicine, Minneapolis, Minnesota, United States of America; 2 Division of Infectious Diseases & International Medicine, Department of Medicine, University of Minnesota Twin Cities School of Medicine, Minneapolis, Minnesota, United States of America; 3 Center for Immunology, Department of Microbiology and Immunology, University of Minnesota Twin Cities School of Medicine, Minneapolis, Minnesota, United States of America; Portland VA Medical Center, Oregon Health and Science University, UNITED STATES OF AMERICA

## Abstract

Although lung myeloid cells provide an intracellular niche for *Mycobacterium tuberculosis* (*Mtb*), CD4^+^ T cells limit *Mtb* growth in these cells to protect the host. The CD4^+^ T cell activities including interferon-γ (IFN-γ) production that account for this protection are poorly understood. Using intravenous antibody labeling and lineage-tracing reporter mice, we show that monocyte-derived macrophages (MDMs), rather than phenotypically similar monocytes or dendritic cells, are preferentially infected with *Mtb* in murine lungs. MDMs were recruited to the lungs by *Mtb-*specific CD4^+^ T cells via IFN-γ, which promoted the extravasation of monocyte precursors from the blood. It was possible that CD4^+^ T cells recruited infectable MDMs because these cells are uniquely poised to receive cognate MHCII-mediated help to control intracellular bacteria. Mice with MHCII deficiency in monocyte-derived cells had normal *Mtb*-specific CD4^+^ T cell activation, expansion and differentiation but the CD4^+^ T cells were unable to attenuate *Mtb* growth. Using single cell RNA sequencing, we showed that MDMs receiving cognate MHCII-mediated help from CD4^+^ T cells upregulated glycolytic genes associated with *Mtb* control. Overall, the results indicate that CD4^+^ T cells recruit infectable MDMs to the lungs and then trigger glycolysis-dependent bacterial control in the MDMs by engaging their MHCII-bound *Mtb* peptides. Moreover, the results suggest that cognate MHCII-mediated help to promote MDM glycolysis is an essential, IFN-γ-independent effector function of *Mtb*-specific CD4^+^ T cells.

## Summary

Tuberculosis, a pulmonary infection caused by the intracellular bacterial pathogen *Mycobacterium tuberculosis* (*Mtb*), is the leading cause of human death by an infectious disease. In most immunocompetent individuals, however, CD4^+^ T cells generate sufficient immunity to prevent active *Mtb* disease. We sought to understand the mechanism of this protection using a mouse model of tuberculosis. We found that CD4^+^ T cells recruited macrophages to the lungs that rapidly took up *Mtb* bacteria. Although the CD4^+^ T cells increased the number of infectable cells in the lungs, they then recognized *Mtb* peptides presented by major histocompatibility complex II (MHCII) molecules on the surface of the infected macrophages and promoted their disinfection. Macrophages that interacted with CD4^+^ T cells in this manner appeared to upregulate glycolysis, a metabolic state required for restriction of intracellular bacteria. Thus, our study suggests that CD4^+^ T cells attempt to clear *Mtb* from the lungs through a “catch and kill” strategy of recruiting infectable macrophages and disinfecting them through cognate MHCII-mediated interactions.

## Introduction

*Mycobacterium tuberculosis* (*Mtb*) is a phagosomal bacterial pathogen that infects the lungs and is responsible for tuberculosis. Most immunocompetent individuals generate sufficient immunity to prevent disease [1,2]. This immunity depends on CD4^+^ T cells as evidenced by the fact that people and primates with CD4^+^ T cell deficiency due to immunodeficiency virus infection, and mice genetically deficient or depleted of CD4^+^ T cells, are hyper-susceptible to tuberculosis [3–7]. The CD4^+^ T cells that control *Mtb* express T cell antigen receptors (TCRs) that are specific for *Mtb* peptides presented by MHC class II molecules on host cells.

In humans, *Mtb* infection induces a mixed population of CD4^+^ T cells consisting of IFN-γ-producing Th1 and IFN-γ- and IL-17-producing Th1* cells [8]. Humans with genetic deficiencies in IFN-γ signaling or IL-12, a cytokine required for Th1 cell differentiation, succumb to lethal infections by environmental mycobacteria as well as the attenuated *Mycobacterium bovis* strain BCG [3,9]. Th1 cells also dominate the *Mtb*-specific CD4^+^ T cell response in C57BL/6 mice [10,11], the most widely used animal model of tuberculosis. Wild-type (WT) C57BL/6 mice develop chronic *Mtb* infections and survive for a year or more, while mice that lack CD4^+^ T cells or have germline deficiencies in IL-12 or IFN-γ signaling experience uncontrolled *Mtb* growth and early lethality [12,13]. Thus, Th1 cells are critical for preventing lethal tuberculosis in humans and mice.

However, the mechanism that Th1 cells use to control *Mtb* infection is unclear. Most notably, IFN-γ production is not sufficient to explain CD4^+^ T cell-mediated protection against tuberculosis. In humans, despite the essentiality of IL-12 and IFN-γ for resistance to BCG and environmental mycobacteria, there is a weaker correlation between IL-12 or IFN-γ signaling defects and lethal *Mtb* infections [3,9]. In mice, IFN-γ-deficient CD4^+^ T cells are only partially defective in restricting pulmonary *Mtb* growth compared to wild type (WT) CD4^+^ T cells [14–19]. CD4^+^ T cells that are deficient in other Th1 products such as TNF-α, perforin, FAS, or GM-CSF are also fully capable of restricting *Mtb* replication in mice [14,16,18,20]. Thus, although there is no doubt that CD4^+^ T cells are critical for control of *Mtb* growth, their mechanism of action has not been resolved.

Prior observations support the notion that CD4^+^ T cells expressing *Mtb* peptide:MHCII-specific TCRs must directly engage infected cells displaying these complexes to control infection. A study of tuberculosis in chimeric mice containing MHCII^+^ and MHCII^–^ cells found that MHCII^–^ cells contained more *Mtb* bacilli than MHCII^+^ cells from the same animal, a difference that was abrogated by CD4^+^ T cell depletion [21]. The cells in which the infection was controlled expressed markers of macrophages or dendritic cells (DCs) and are variously referred to as interstitial macrophages (IMs) [22,23], monocyte-derived or recruited macrophages [21,24–26], or DCs [21,27]. The true ontogeny of IMs has thus been debated. In C57BL/6 mice infected with *Mtb*, IMs are distinguished from other tissue-resident macrophages in part by a high degree of glycolytic metabolism [23,28,29]. The propensity of IMs to perform glycolysis may be critical for restricting *Mtb* growth and protecting hosts from lethal disease because mice with myeloid cell-specific deficiency in the main positive regulator of glycolysis, hypoxia inducible factor 1 alpha (HIF-1α), are hyper-susceptible to tuberculosis [30]. It is possible that CD4^+^ T cells induce IM glycolysis by recognizing *Mtb* peptide:MHCII complexes on their surface and providing an activation signal. Supporting this possibility, this form of cognate CD4^+^ T cell help to B cells increases glucose uptake and activation of the pro-glycolytic regulators mTORC1 and c-Myc, which are critical for germinal center selection [31–33].

The contribution of cognate CD4^+^ T cell help to protection against tuberculosis not been assessed in definitive way because the ontogeny of the IMs that phagocytose *Mtb* has not been conclusively demonstrated, precluding approaches to selectively ablate MHCII molecules in IMs while preserving antigen presentation to CD4^+^ T cells in secondary lymphoid tissues. In this study, we used lineage-tracing reporter mice to demonstrate that IMs within *Mtb*-infected lungs are almost entirely of monocyte origin, and thus identify them as monocyte-derived macrophages (MDMs). We found that CD4^+^ T cells recruit infectable MDMs to the lungs by producing IFN-γ but then recognize MHCII-bound *Mtb* peptides on these cells, triggering MDM glycolysis and bacterial control via an IFN-γ-independent mechanism. The ability of CD4^+^ T cells to restrict *Mtb* growth in the lungs was entirely dependent on MHCII expression by monocyte-derived cells, indicating that cognate CD4^+^ T cell help to MDMs is an essential IFN-γ-independent activity in tuberculosis immunity.

## Results

### Monocyte-derived macrophages (MDMs) are preferentially infected with *Mtb*

A low dose (~100 bacilli/mouse) aerosol infection of C57BL/6 mice was chosen because CD4^+^ T cells restrict *Mtb* growth and protect from lethality under these conditions [6,34]. Experiments were first performed to clarify the identity and ontogeny of lung myeloid cells during *Mtb* infection as a prerequisite for studying their regulation by CD4^+^ T cells. A major goal was to improve on prior analyses of lung myeloid cell subsets [22–25,27] by more accurately discriminating lung-resident cells from contaminating blood cells. To that end, CX_3_CR1 staining was used to distinguish monocytes, which were expected to primarily reside in blood, from lung-resident macrophages and DCs. Mice were also administered intravenous (i.v.) CD45 antibody 3 minutes prior to sacrifice to label cells in the blood [35].

**Fig 1:**
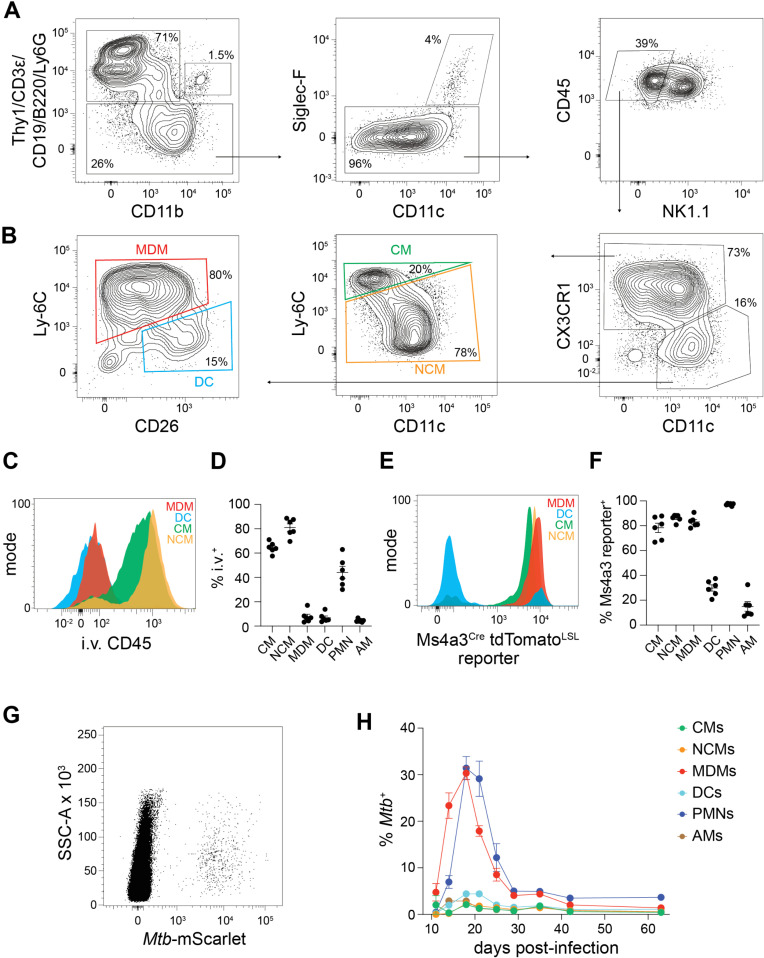
CX3CR1^lo^ CD11c^hi^ CD26^lo^ Ly-6C^hi^ monocyte-derived macrophages (MDMs) are preferentially infected with *Mtb.* (A-B) Cells in the lungs of C57BL/6 mice at 3 weeks post-infection with *Mtb*, pre-gated on CD45^+^ singlets. MDM, monocyte-derived macrophage; DC, dendritic cell; CM, classical monocyte; NCM, nonclassical monocyte. (C-D) Staining of indicated lung populations with intravascular (i.v.) CD45 antibody indicating blood residence (see Materials and Methods). PMN, neutrophil; AM, alveolar macrophage. Symbols represent biological replicates (n = 6 per group) with the group mean ± standard error of the mean (SEM). (E-F) tdTomato fluorescence among indicated cell populations. Symbols represent biological replicates (n = 6 per group) with the group mean ± SEM. (G) CD45^+^ cells from the lungs of mice infected with *Mtb*-mScarlet. (H) Frequency of *Mtb*-mScarlet-infected cells within each of the indicated lung populations. Data at each time point represents n = 4 biological samples collected from 2 independent experiments with the group mean ± SEM. Data for (A-G) were collected at 3 weeks post-infection.

Lung single cell suspensions were analyzed at 3 weeks post-infection using flow cytometry. After excluding T and B cells, canonical markers were used to identify neutrophils (PMNs; Ly-6G^+^ CD11b^+^), alveolar macrophages (AMs; SIGLEC-F^+^ CD11c^+^), and NK cells (NK1.1^+^) (Fig 1A). Among the remaining cells, a large population of CX_3_CR1^hi^ CD11c^—/lo^ cells and a smaller population of CX3CR1^lo^ CD11c^hi^ cells were detected. The CX_3_CR1^hi^ CD11c^—/lo^ cells contained two subsets: Ly-6C^hi^ CD11c^—^ classical monocytes (CMs) and Ly-6C^lo^ CD11c^lo^ nonclassical monocytes (NCMs) (Fig 1B), both of which were predominantly in the blood based on i.v. labeling with CD45 antibody (Fig 1C–D). The Ly-6C^lo^ CD11c^lo^ cells were confirmed to be NCMs based on TremL4 positivity (S1A Fig) [36]. The CX3CR1^lo^ CD11c^hi^ population could also be divided into two subsets: Ly-6C^hi^ CD26^lo^ cells, provisionally identified as monocyte-derived macrophages (MDMs), and Ly-6C^lo^ CD26^hi^ cells, provisionally identified as DCs (Fig 1B). Consistent with these putative identities, MHCII and CD64 staining was lower on the CMs and NCMs than on the MDMs and DCs (S1B Fig) and most of the MDMs and DC were in the lung tissue based on a lack of i.v. labeling with CD45 antibody (Fig 1C-D). Many PMNs were also labeled with i.v. CD45 antibody while AMs were not, consistent with their location in blood and tissue, respectively (Fig 1D). Thus, the flow cytometry gating strategy (Fig 1A-B) effectively discriminated lung-resident MDMs and DCs from more numerous, primarily blood-restricted monocytes.

The identity and ontogeny of the MDMs was further tested using lineage-tracing reporter mice. *Ms4a3* is expressed by bone marrow cells of the monocyte and granulocyte lineages, but not in classical DCs (cDCs) or non-hematopoietic macrophages such as AMs [37]. *Ms4a3*^*Cre*^ *tdTomato*^*LSL*^ mice, in which cells of monocyte or granulocyte origin express tdTomato, were infected with *Mtb* and assayed at 3 weeks post-infection to assess the origins of the lung myeloid populations. tdTomato was expressed by 80–90% of MDMs, CMs and NCMs, but only 30% of DCs (Fig 1E–F). Thus, the cells that were identified as MDMs by the flow cytometry strategy shown in Fig 1A**–**B arose from the monocyte lineage. The observation that some DCs were tdTomato^+^ indicated that a small number of DCs are monocyte-derived or that the *Ms4a3* reporter is expressed in an off-target fashion in some cDCs. Most AMs were tdTomato^—^, reflecting their non-hematopoietic origin, while all PMNs were tdTomato^+^ (**Fig 1F**).

The cell identification scheme shown in Fig 1A, 1B and a strain of *Mtb* H37Rv expressing the fluorescent protein mScarlet (*Mtb*-mScarlet) were used to determine which lung cells contained *Mtb* bacteria after infection (**Fig 1G**). Approximately 20% of MDMs and 30% of PMNs contained *Mtb* 3 weeks after infection. The percentage of infected MDMs increased dramatically between days 11 and 14, peaked on day 18 at 30%, fell to 5% by day 30, and was maintained at 5% until day 63. The percentage of infected PMNs followed a similar rise and fall but was delayed about 2 days compared to the MDMs. Less than 5% of CMs, NCMs, DCs, or AMs contained bacteria at any time (**Fig 1H**).

Overall, the i.v. labeling, lineage tracing and phenotyping results demonstrated that CX_3_CR1, CD11c, Ly-6C and CD26 resolve CMs, NCMs, MDMs and DCs and account for most of the myeloid cells in lung single cell suspensions from *Mtb*-infected mice. This strategy is an advance because it discriminates lung-resident MDMs from DCs and blood-resident NCMs, while earlier studies could not resolve these populations based on CD11c, MHCII and CD64 expression. This flow cytometry strategy also demonstrated that MDMs, rather than DCs, monocytes, or non-monocyte-derived macrophages, are the primary non-granulocytic cell population that becomes infected with *Mtb*.

### CD4^+^ T cells recruit MDMs by promoting the migration of CM precursors into the lungs

**Fig 2:**
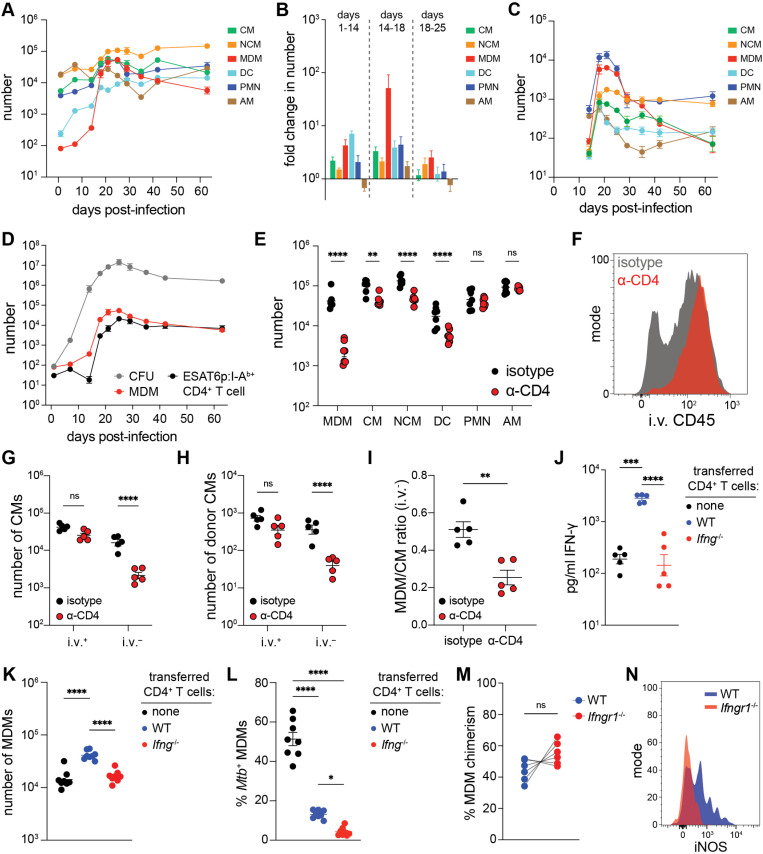
CD4 ^**+**^ **T cells recruit MDMs to the lungs via IFN-γ**. (A) Number of indicated cells in the lungs of *Mtb*-infected mice. Cell numbers were calculated by flow cytometry using counting beads (see Materials and Methods). (B) Fold change in cell count for each population in the indicated time intervals. Values were calculated using the data from (A) by dividing the number of cells at the later time point by the group mean of cells at the earlier time point. (C) Number of *Mtb*^+^ cells among the indicated populations in the lungs of mice based on the number of cells positive for the *Mtb*-derived mScarlet fluorescent protein. (D) Number of *Mtb* colony-forming units (CFUs), MDMs and ESAT6 peptide-specific CD4^+^ T cells (based on staining with fluorescently-labeled I-A^b^ tetramer containing a peptide from the *Mtb* protein ESAT6, ESAT6p:I-A^b^) in the lungs of infected mice. (E) Number of indicated cells in the lungs of mice treated with CD4^+^ T cell-depleting antibody (α-CD4) or isotype control antibody. Antibody treatment was performed on day 0, 7 and 14 post-infection and the data was collected at 3 weeks post-infection. (F) Staining with i.v. CD45 antibody (indicating location in blood) among the CMs from the lungs of mice treated with the indicated antibodies. (G) Number of i.v. CD45 antibody-positive and -negative CMs in the lungs of mice treated with indicated antibodies. (H) Number of i.v. CD45 antibody-positive and -negative, congenically marked donor CMs in the lungs of *Mtb*-infected recipient mice 18 hours after adoptive transfer. (I) Ratio of MDMs to CMs among total i.v.^–^ MDMs and CMs. (J) IFN-γ concentration in lung homogenates from T cell-deficient *Tcra*^–/–^ mice containing adoptively transferred CD4^+^ T cells from infection-naive WT or *Ifng*^–/–^ donor mice or containing no transferred CD4^+^ T cells. (K) Number of MDMs and (L) frequency of *Mtb*^+^ MDMs based on mScarlet positivity in the lungs of mice as described in (J). (M) Bone marrow origin (chimerism) of lung MDMs in mice containing a 50/50 mix of WT (CD45.1^+^) and *Ifngr1*^–/–^ (CD45.1^–^) bone marrow. (N) iNOS antibody positivity among WT and *Ifngr1*^–/–^ MDMs from (L). Data was collected at 3 weeks (E-I) or 4 weeks (J-M) post-infection. Data represents n = 4 per time point (A-D), n = 7 (E), n = 5 (G-I), n ≥ 5 (J-L), and n = 6 (M-N) biological samples each from 2 independent experiments per group with the group the mean ± SEM. Statistical significance was determined by two-way ANOVA with Šidák correction (E, G, H), unpaired t test with Welch’s correction (I), one-way ANOVA with Tukey correction (J-L), and Wilcoxon matched-pairs signed rank test (M). *p < 0.05, **p < 0.01, ***p < 0.01, ****p < 0.0001.

The kinetics of myeloid cell recruitment to the lungs during *Mtb* infection, and the role of CD4^+^ T cells in this process, was then determined. The numbers of all hematopoietic myeloid populations (MDMs, CMs, NCMs, PMNs, and DCs) increased during the first 3 weeks of infection and remained stable or gradually waned by week 9 (**Fig 2A**). By contrast, the abundance of AMs, which are largely of non-hematopoietic origin at 3 weeks post-infection (**Fig 1F**), remained similar between naïve and 9 week-infected mice (**Fig 2A**).

MDMs exhibited the greatest degree of recruitment to the lungs. Only about 100 MDMs were present on day 1 post infection. The number went up to several hundred by day 14, but then to over 10,000 by day 18 (**Fig 2A**), increasing nearly 100-fold in that interval (**Fig 2B**). By contrast, CMs, NCMs, DCs and PMNs all increased less than 10-fold at all time periods analyzed (**Fig 2B**). Together, the results indicated that most MDMs are recruited to the lungs between 14 and 18 days post-infection while the abundance of CMs, NCMs, PMNs and DCs increased more slowly and consistently. Importantly, the number of *Mtb*-infected MDMs also increased dramatically between days 14 and 18 such that MDMs became the major infected population along with PMNs (**Fig 2C**). Thus, recruited MDMs were a major reservoir of infection.

The sharp increase in lung MDMs corresponded to the time when *Mtb*-specific CD4^+^ T cells have been reported to migrate into the lungs [38]. This was assessed by measuring lung CD4^+^ T cells that expressed TCRs that bound to a fluorescently-labeled I-A^b^ (the MHCII molecule of C57BL/6 mice) tetramer containing a peptide from the *Mtb* protein ESAT6 (ESAT6p:I-A^b^) [39] (S2A Fig). The numbers of MDMs and *Mtb* colony-forming units (CFUs), which reflects the total number of bacilli in the lungs, were additionally measured. Although the number of *Mtb* bacteria rose to nearly 1 million by day 14, few ESAT6p:I-A^b^ tetramer-binding CD4^+^ T cells or MDMs were detected at this time. By day 18, however, the number ESAT6p:I-A^b^ tetramer-binding CD4^+^ T cells and MDMs had risen approximately 100-fold. The ESAT6p:I-A^b+^ CD4^+^ T cells and MDMs both peaked on day 25 and tapered slowly through day 63. The *Mtb* CFUs also peaked on day 25 and declined about 10-fold by day 42 (Fig 2D). The reduction in *Mtb* CFUs occurred more slowly than the reduction in infected myeloid cells based on flow cytometry, which peaked on day 21 and declined about 10-fold by day 28 (Fig 2C). The difference in kinetics may reflect more rapid clearance of *Mtb* from cells containing lower numbers of bacilli or the presence of extracellular bacilli released by dying host cells. Nevertheless, this experiment confirmed that *Mtb*-specific CD4^+^ T cells and MDMs are recruited to the lungs in the same time frame.

The hypothesis that CD4^+^ T cells were responsible for the recruitment of lung MDMs was next tested by treating mice with a CD4^+^ T cell-depleting (α-CD4) or isotype control antibody and then infecting the treated mice with *Mtb*. No CD4^+^ T cells were observed in the lungs of α-CD4-treated mice at week 3 post-infection demonstrating that the antibody depletion was effective (S2B Fig). Approximately 20-fold fewer MDMs were observed in lung single cell suspensions of CD4^+^ T cell-depleted mice at this time compared to non-depleted mice. By contrast, the numbers of CMs, NCMs, or DCs were only 2- to 3-fold lower in CD4^+^ T cell-depleted mice, while the numbers of PMNs and AMs were not affected. (Fig 2E). The CD4 antibody was unlikely to deplete MDMs directly, as MDMs did not bind the CD4 antibody used for depletion (S2C Fig). The difference in MDM abundance could not be accounted for by differences in *Mtb* CFUs, which as expected were comparable between CD4^+^ T cell-deficient and -sufficient mice at this time (S2D Fig) [6]. Thus, CD4^+^ T cells are responsible for the majority of MDM recruitment to the lungs during the first 3 weeks of infection.

The i.v. labeling experiments demonstrated that approximately 35% of CMs and all MDMs were lung resident (**Fig 1D**). This result raised the possibility that CD4^+^ T cells recruit CMs from the blood into the lung tissue, where the CMs then differentiate into MDMs. Intravenous CD45 antibody labeling was performed on α-CD4- or isotype control antibody-treated mice at 3 weeks post-infection to test this possibility. Isotype control antibody- and α-CD4-treated mice had equal numbers of i.v. CD45 antibody^+^ CMs in the blood. Strikingly, however, α-CD4-treated mice had 8-fold fewer i.v. CD45 antibody^–^ lung-resident CMs compared to isotype control antibody-treated mice (Fig 2F**–**G) indicating that CD4^+^ T cells promoted the extravasation of blood CMs into the lungs.

A CM transfer experiment was then performed to validate this conclusion. CMs were purified from the bone marrow of naïve CD45.1^+/–^ donor mice and injected into the blood of *Mtb*-infected recipient mice that had been treated with α-CD4 or isotype control antibody (S2E Fig). The location of the donor cells in the lungs was monitored 18 hours after injection. About 7-fold fewer donor cells became i.v. CD45 antibody^–^ lung-resident in α-CD4-treated compared to isotype control antibody-treated mice, while both groups of mice contained a similar number of donor i.v. CD45 antibody^+^ cells in the blood (Fig 2H). Overall, the results demonstrated that CD4^+^ T cells recruit MDMs by promoting the migration of the MDM precursors, CMs, into the lungs.

It was possible that CD4^+^ T cells also induced the differentiation of lung-resident CMs into MDMs. To account for this possibility, the ratio of MDMs to CMs was calculated within the i.v. CD45 antibody^–^ fraction of the lung single cell suspensions. The ratio of MDMs to CMs was approximately 2-fold lower in CD4^+^ T cell-depleted mice compared to intact control mice (**Fig 2I**). Thus, the 20-fold decrease in MDMs observed in CD4^+^ T cell-depleted mice was explained primarily by the failure of CM precursors to extravasate into the lungs, but also by a decrease in the rate at which lung-resident CMs differentiate into MDMs.

### MDM recruitment, but not *Mtb* control, is mediated by CD4^+^ T cell-derived IFN-γ

The role of CD4^+^ T cell-derived IFN-γ in lung MDM recruitment was explored because most *Mtb-*specific CD4^+^ T cells are Th1 cells [10,11]. The abundance of IFN-γ in the lungs was 10-fold lower in CD4^+^ T cell-depleted compared to isotype antibody-treated mice at 3 weeks post-infection (S2F Fig), consistent with prior reports [6]. To test if CD4^+^ T cell-derived IFN-γ contributed to MDM recruitment, mice were generated with or without the *Ifng* gene in CD4^+^ T cells by transferring CD4^+^ T cells from naïve WT or *Ifng*^—*/*—^ donor mice into T cell-deficient (*Tcra*^—*/*—^) recipients. Both groups of recipients, as well as a control group of T cell-deficient mice receiving no transferred cells, were then infected with *Mtb* to assess IFN-γ levels and MDM abundance in the lungs. At 4 weeks post-infection, T cell-deficient mice that received WT donor CD4^+^ T cells contained significantly more lung IFN-γ than mice that received IFN-γ-deficient CD4^+^ T cells or no CD4^+^ T cells (Fig 2J). Moreover, T cell-deficient mice that received WT CD4^+^ T cells contained significantly more lung MDMs than T cell-deficient mice that received no CD4^+^ T cells, and this increase in MDMs was completely absent in T cell-deficient mice that received IFN-γ-deficient CD4^+^ T cells (Fig 2K). In contrast, the transferred WT and IFN-γ-deficient CD4^+^ T cells restricted *Mtb* growth within MDMs equally well based on the frequency of MDMs that contained *Mtb* (Fig 2L). Likewise, compared to mice with no T cells, the mice with WT and IFN-γ-deficient CD4^+^ T cells had equally low frequencies of infected CMs, NCMs, DCs, PMNs and AMs (S2G Fig) and total *Mtb* bacilli in the lungs S2H Fig). Expression of MHCII, which can be induced by IFN-γ [40], was similarly high in MDMs and DCs from all three groups of mice although a slight reduction was observed in mice with IFN-γ-deficient CD4^+^ T cells (S2I Fig). These results were consistent with prior reports that the antimicrobial effect of *Mtb*-specific CD4^+^ T cells cannot be fully explained by their production of IFN-γ [14–19,41]. Thus, CD4^+^ T cells recruit MDMs by secreting IFN-γ but control *Mtb* growth within the MDMs through another mechanism.

It was next determined if CMs must undergo IFN-γ signaling to migrate into the lungs and become MDMs. Mixed bone marrow chimeric mice were generated by reconstituting irradiated WT mice with a 1:1 ratio of WT (CD45.1^+^) and IFN-γ receptor 1 (IFNγR1)-deficient (*Ifngr1*^—*/*—^) (CD45.2^+^) bone marrow cells. The chimeric mice were infected with *Mtb* and MDMs were identified in the lungs at 3 weeks post-infection (S2J Fig). Unexpectedly, there were similar if not more MDMs derived from the IFNγR1-deficient compared to WT bone marrow cells (Fig 2M). By contrast, inducible nitric oxide synthase (iNOS), which is induced by IFN-γ receptor signaling and promoted by CD4^+^ T cells [6,42], was only detected in MDMs derived from WT bone marrow cells (Fig 2N). Thus, although CD4^+^ T cell-derived IFN-γ promoted the accumulation of MDMs in the lungs, expression of the IFN-γ receptor by the MDMs or their precursors was not required for this activity. Instead, CD4^+^ T cell-derived IFN-γ must promote MDM recruitment indirectly by signaling to other cells. Importantly, these experiments reveal that MDM recruitment and MDM expression of IFN-γ-inducible factors such as iNOS are two temporally distinct, IFN-γ mediated activities promoted by CD4^+^ T cells.

### CD4^+^ T cell-mediated attenuation of *Mtb* growth requires antigen presentation by CCR2^+^ cells

Our results indicated that *Mtb*-specific CD4^+^ T cells recruit infectable MDMs via IFN-γ but inhibit or kill *Mtb* within the recruited MDMs through a IFN-γ-independent mechanism (Figs 2L and S2H Fig) as shown in other studies [14,16–18]. It was possible that CD4^+^ T cells triggered *Mtb* control in MDMs by recognizing MHCII-bound *Mtb* peptides on the MDM surface. This possibility was addressed using *CCR2*^*CreERT2-GFP/WT*^ *H2-Ab1*^*flox/flox*^ (CCR2^ΔMHCII^) mice in which tamoxifen (TAM) treatment causes cells expressing CCR2, such as CMs, NCMs, and MDMs, to delete the *H2-Ab1* gene encoding the I-A^b^ beta chain of MHCII [43].

**Fig 3:**
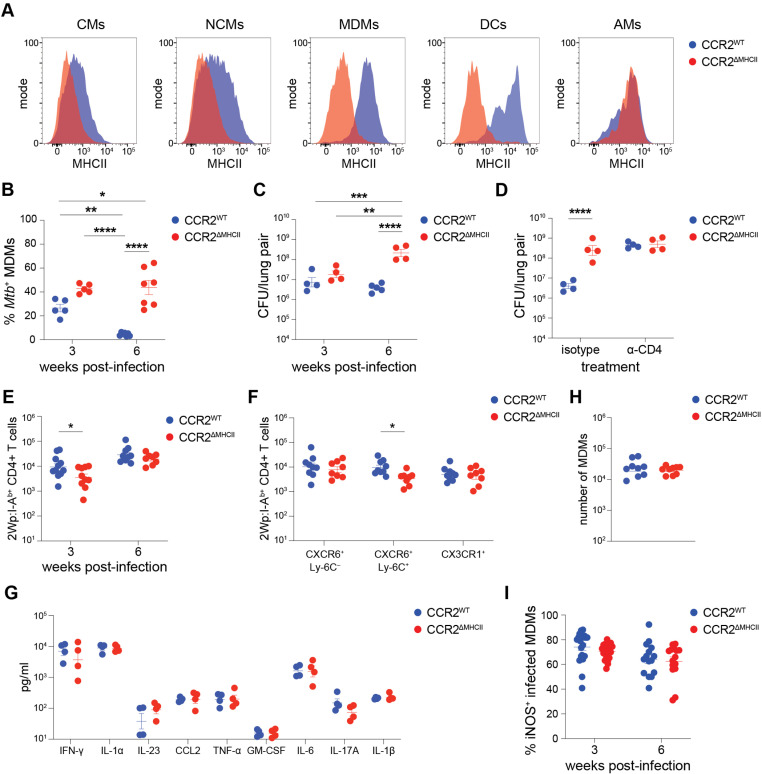
CD4 ^**+**^ **T cell control of *Mtb* requires MHCII expression by CCR2**^**+**^ **cells.** (A) MHCII expression among indicated lung populations from tamoxifen-fed CCR2^CreERT2-GFP/WT^ *H2-Ab1*^flox/flox^ (CCR2^ΔMHCII^) and Cre^—^ *H2-Ab1*^flox/flox^ littermates (CCR2^WT^) at 3 weeks post-infection. Data are representative of ≥ 5 independent experiments. (B) Frequency of lung MDMs containing *Mtb* (based on positivity for the *Mtb*-derived mScarlet fluorescent protein) and (C) *Mtb* CFUs in the lungs of indicated mice. (D) *Mtb* CFUs at 6 weeks post-infection in the lungs of indicated mice treated with isotype or CD4^+^ T cell-depleting antibodies weekly starting on the day of infection. (E) Number of 2W peptide-specific CD4^+^ T cells (based on staining with fluorescently-labeled I-A^b^ tetramer containing 2W peptide expressed by *Mtb*, 2Wp:I-A^b^) in the lungs of indicated mice. (F) Number of CXCR6^+^ Ly-6C^–^ Th1 resident memory, CXCR6^+^ Ly-6C^+^ Th1 effector memory, and terminally differentiated CX_3_CR1^+^ Th1 cells [10,47–49] among the 2Wp:I-A^b^ tetramer^+^ CD4^+^ T cells from indicated mice at 6 weeks post-infection. (G) Abundance of indicated cytokines in lung homogenates from indicated mice at 3 weeks post-infection. (H) Number of MDMs in the lungs of indicated mice at 3 weeks post-infection. (I) Frequency iNOS positivity based on intracellular antibody staining among infected (*Mtb*-mScarlet^+^) MDMs in the lungs of indicated mice. Symbols represent n ≥ 5 (A-B), n = 4 (C-D), n ≥ 7 (E-F), n = 4 (G) and n ≥ 9 (H-I) biological samples from at least 2 independent experiments per group with the group mean ± SEM indicated. Statistical significance was determined by two-way ANOVA with Šidák correction (B-G, I) and unpaired t test with Welch’s correction (H). *p < 0.05, **p < 0.01, ****p < 0.0001.

CCR2^ΔMHCII^ and Cre^–^ littermates (CCR2^WT^) were infected with *Mtb* and given TAM-containing chow starting on the day of infection. At 3 weeks post-infection, the CMs, NCMs, and MDMs in the lungs of CCR2^ΔMHCII^ mice lacked MHCII as expected. The DCs in the lungs of CCR2^ΔMHCII^ mice also lacked MHCII while MHCII expression on AMs was preserved (**Fig 3A**). The observation that MHCII was absent from lung DCs in CCR2^ΔMHCII^ mice is consistent with prior observations that cDCs are recruited from the bone marrow to the lungs in a CCR2-dependent manner during infection [44,45].

TAM-treated CCR2^WT^ and CCR2^ΔMHCII^ mice were infected with *Mtb*-mScarlet and the frequency of infected MDMs was compared to test if antigen presentation by MDMs to CD4^+^ T cells is required for *Mtb* control. In the lungs of CCR2^WT^ mice, 25% of the MDMs contained *Mtb* bacteria at 3 weeks post-infection and this frequency declined to 5% by 6 weeks post-infection (Fig 3B) as observed in earlier experiments (Fig 1H). In contrast, 40% of MDMs in CCR2^ΔMHCII^ mice contained *Mtb* bacteria at 3 weeks and the frequency did not decline by 6 weeks post-infection. (Fig 3B). A greater frequency of infected CMs, NCMs, DCs, PMNs and AMs was also observed in CCR2^ΔMHCII^ mice at 6 weeks post-infection (S3A Fig), likely reflecting uncontrolled *Mtb* growth in the lungs at this time. Supporting this conclusion, at 3 weeks post-infection, CCR2^WT^ and CCR2^ΔMHCII^ mice had a similar *Mtb* CFU burden but by 6 weeks post-infection, there were nearly 100-fold more bacteria in the lungs of CCR2^ΔMHCII^ than CCR2^WT^ mice (Fig 3C). Thus, *Mtb* control was highly attenuated when MHCII was absent from cells with a history of CCR2 expression.

The extent to which CD4^+^ T cell-mediated protection was lost in CCR2^ΔMHCII^ mice was measured by treating CCR2^WT^ and CCR2^ΔMHCII^ mice with CD4^+^ T cell depleting or isotype control antibodies. In CCR2^WT^ mice, CD4^+^ T cell depletion resulted in a 100-fold increase in *Mtb* bacteria within the lungs at 6 weeks post-infection. In CCR2^ΔMHCII^ mice, by contrast, CD4^+^ T cell depletion had no effect on the lung *Mtb* burden, which was comparable to CD4^+^ T cell-depleted CCR2^WT^ mice (**Fig 3D**). Overall, the results demonstrated that CD4^+^ T cells are unable to provide any degree of protection against *Mtb* growth in the lungs when MHCII is absent from cells with a history of CCR2 expression.

### *Mtb*-specific Th1 cells develop normally in CCR2^ΔMHCII^ mice despite their inability to restrict *Mtb*

It was possible that the failure of CCR2^ΔMHCII^ mice to restrict *Mtb* growth was due to a defect in the activation, differentiation, or expansion of *Mtb*-specific CD4^+^ T cells. This possibility was tested by tracking CD4^+^ T cells specific for a model peptide antigen called 2W [46] in mice infected with an *Mtb* strain engineered to express this peptide under the control of the constitutive *hsp60* promoter (S3B Fig). While CCR2^ΔMHCII^ mice contained slightly fewer 2W:I-A^b^-specific CD4^+^ T cells in the lungs than CCR2^WT^ mice at 3 weeks post-infection, this difference was no longer observed by 6 weeks post-infection (Fig 3E). In addition, the *Mtb*-specific CD4^+^ T cell population in *Mtb*-infected CCR2^ΔMHCII^ mice contained the same number of CXCR6^+^ Ly-6C^–^ Th1 resident memory and terminally differentiated CX_3_CR1^+^ Th1 cells, and only marginally fewer CXCR6^+^ Ly-6C^+^ Th1 effector memory cells [10,47–49] than CCR2^WT^ mice at 6 weeks post-infection (Fig 3F). CCR2^ΔMHCII^ mice therefore had no defects in *Mtb*-specific CD4^+^ T cell expansion, differentiation, or lung migration that could explain the complete loss of CD4^+^ T cell-mediated protection in these mice (Fig 3D).

While only a minor and transient decrease in the number of *Mtb*-specific Th1 cells was observed in CCR2^ΔMHCII^ mice, it was possible that TCR stimulation by *Mtb* peptide:MHCII complexes on cells with a history of CCR2 expression was required for the production of antimicrobial cytokines. To account for this possibility, a panel of inflammatory cytokines was assayed in the lungs of TAM-treated CCR2^WT^ and CCR2^ΔMHCII^ mice at 3 weeks post-infection, a time at which *Mtb* burden is similar between both groups of mice (Fig 3C). There were no differences in the abundance of IFN-γ, IL-1α, IL-23, CCL2, TNF-α, GM-CSF, IL-6, IL-17A or IL-1β between CCR2^WT^ and CCR2^ΔMHCII^ mice (Fig 3G). As a control, the same assay was performed on WT mice treated with CD4^+^ T cell-depleting or isotype control antibodies. CD4^+^ T cell-depleted mice had significantly lower levels of IFN-γ, IL-1α, CCL2, IL-6 and IL-1β compared to CD4^+^ T cell-replete mice (S3C Fig). It was also noted that the lungs of CCR2^WT^ and CCR2^ΔMHCII^ mice contained similar numbers of MDMs (Fig 3H), the recruitment of which is dependent on IFN-γ-secreting CD4^+^ T cells (Fig 2K). Finally, a similar frequency of infected MDMs stained positively for the IFN-γ-inducible protein iNOS (Fig 2N) in CCR2^WT^ and CCR2^ΔMHCII^ mice at 3- and 6-weeks post-infection (Fig 3I). Overall, the results showed that MHCII antigen presentation by cells with a history of CCR2 expression is dispensable for the production of *Mtb*-specific Th1 cells and cytokines, including IFN-γ, but required for Th1 cells to halt *Mtb* growth in the lungs.

### CMs, NCMs and DCs, but not MDMs, require cognate interactions for optimal IFN-γ receptor signaling

Transcriptional analysis was then performed to search for the basis of MHCII-dependent antimicrobial effects in cells with a history of CCR2 expression. Single cell RNA sequencing was conducted on lymphocyte-, neutrophil-, and AM-depleted populations from the lungs of TAM-fed CCR2^WT^ and CCR2^ΔMHCII^ mice 3 weeks post-infection, a time when *Mtb* burden was similar between the two groups (Figs 3B**–**C).

**Fig 4:**
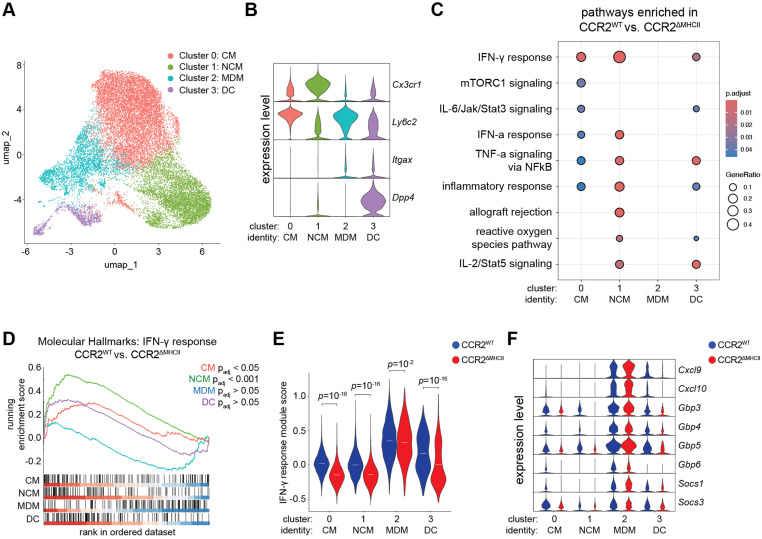
CMs, NCMs and DCs, but not MDMs, require cognate interactions with CD4 ^**+**^ **T cells for IFN-γ** **signaling.** (A) Clustering of combined lung myeloid cell populations based on single cell RNA sequencing of lung cells from CCR2^WT^ and CCR2^ΔMHCII^ mice at 3 weeks post-infection. (B) Relative expression of indicated genes (right) among myeloid cell clusters (bottom). Full set of cluster-defining genes are presented in S1 Table. (C) For each myeloid cell cluster (bottom), biological pathways enriched within the top differentially expressed genes (log_2_ fold change > 0.5) in CCR2^WT^ mice compared to CCR2^ΔMHCII^ mice. Pathways were identified using over-representation analysis by integrating the mSigDB Molecular Hallmarks geneset collection (see Materials and Methods). (D) Gene set enrichment analysis (GSEA) of the genes ranked by log_2_ fold change that were preferentially expressed in the indicated myeloid cell clusters from CCR2^WT^ mice compared to their respective populations in CCR2^ΔMHCII^ mice, referencing the mSigDB Molecular Hallmarks “response to interferon gamma” geneset. (E) Aggregate expression of IFN-γ-inducible genes (“IFN-γ response module”; see S3D Table) among the indicated myeloid cell clusters from CCR2^WT^ and CCR2^ΔMHCII^ mice. (F) Expression of select IFN-γ-inducible genes in myeloid cell clusters from CCR2^WT^ and CCR2^ΔMHCII^ mice. Statistical significance was determined by hypergeometric test with Benjamini-Hochberg correction (C, E) or rank-based enrichment scoring with Benjamini-Hochberg correction (D). Data was collected from 2 CCR2^WT^ mice (1 male and 1 female) and 2 CCR2^ΔMHCII^ mice (1 male and 1 female).

Analysis of the combined data from CCR2^WT^ and CCR2^ΔMHCII^ mice produced 4 cell clusters (Fig 4A) that corresponded to the CMs, NCMs, MDMs and DCs (Fig 1B) (cluster-defining genes listed in S1 Table). Cells in cluster 0 were CMs based on expression of L-selectin (*Sell*) [50], fibronectin 1 (*Fn1*) and coagulation factor XIII A chain (*F13a1*) [51]. Cluster 1 cells were NCMs based on *Cd36*, *Ace*, and *TremL4* expression [52]. Cells in cluster 2 were MDMs based on expression of chemokine receptors found on inflammatory macrophages (*Cxcl9*, *Cxcl10*) [53] as well as *Ass1*, encoding an arginine biosynthesis enzyme important for nitric oxide production [54]. Lastly, cluster 3 cells were DCs based on expression of *Flt3* and *Zbtb46* [55] (S4A Fig). Consistent with earlier flow cytometry results, CMs (cluster 0) and NCMs (cluster 1) had high *Cx3cr1* and low *Itgax* (encoding CD11c) expression while CMs were enriched for *Ly6c2*. MDMs (cluster 2) had low *Cx3cr1* and high *Itgax* and *Ly6c2* expression, while DCs (cluster 3) uniquely expressed *Dpp4* (CD26) (Fig 4B). As expected, MHCII (*H2-Ab1*) transcripts were absent from CMs, NCMs, MDMs and most DCs among the cells from CCR2^ΔMHCII^ mice compared to the cells from CCR2^WT^ mice (S4B Fig). Thus, flow cytometry and scRNA-seq analysis identified the same 4 classes of lung myeloid cells.

The CMs, NCMs, MDMs and DCs were assessed for genes differentially expressed between CCR2^WT^ and CCR2^ΔMHCII^ mice. There were 145 genes that were expressed by at least 10 percent of cells and had a log_2_ fold change in expression of 0.5 or greater between the CMs of CCR2^WT^ and CCR2^ΔMHCII^ mice. A similar magnitude of differentially expressed genes was observed for NCMs (159), MDMs (141) and DCs (270) (full set of differentially expressed genes in S2A–D Table).

The most highly upregulated genes (log_2_ fold change > 0.5) in cells from CCR2^WT^ compared to CCR2^ΔMHCII^ mice were used to identify biological pathways that were induced in an MHCII-dependent fashion. Over-representation analysis was performed using the Molecular Signatures Database (MSigDB) Molecular Hallmarks collection of gene sets as a reference [56] (S3A–C Table). Among CMs, NCMs and DCs, the most highly enriched biological pathway in CCR2^WT^ compared to CCR2^ΔMHCII^ mice was “interferon gamma response.” The CMs, NCMs and DCs from CCR2^WT^ mice also preferentially expressed genes involved in mTORC1 signaling, IL-6/Jak/Stat3 signaling, IFN-α signaling, TNF-α signaling, inflammatory response, allograft rejection, reactive oxygen species, and IL-2/Stat5 signaling when compared to the CMs, NCMs and DCs from CCR2^ΔMHCII^ mice (Fig 4C). Many of these gene sets contained the same IFN-γ-inducible genes such as *Cxcl10*, *Gbp3*, *Socs1* and *Ifitm* [57–60] (S3A–C Table) indicating that IFN-γ receptor signaling was the most prominent MHCII-dependent feature in CMs, NCMs and DCs. To support this conclusion, gene set enrichment analysis (GSEA) was performed on the differentially expressed genes, ranked by log_2_ fold change, for each cluster. CMs and NCMs, but not DCs, had significant positive enrichments for IFN-γ receptor signaling when comparing cells from CCR2^WT^ and CCR2^ΔMHCII^ mice (Fig 4D). Overall, the over-representation and GSEA analyses suggested that antigen presentation to CD4^+^ T cells is required for optimal IFN-γ receptor signaling in CMs and NCMs, and to a lesser extent DCs.

Unexpectedly, no biological pathways, including IFN-γ receptor signaling, were significantly enriched in the MDMs of CCR2^WT^ mice compared to the MDMs of CCR2^ΔMHCII^ mice (**Fig 4C**). This finding was explored in more detail by creating an “IFN-γ response module” consisting of the IFN-γ-inducible genes that were enriched in CMs from CCR2^WT^ versus CCR2^ΔMHCII^ mice (S3D Table). MDMs expressed the highest IFN-γ response signature, and its expression was slightly lower in CCR2^ΔMHCII^ mice compared to CCR2^WT^ mice. By contrast, CMs, NCMs and DCs had an overall lower IFN-γ response signature that was strongly attenuated in the cells from CCR2^ΔMHCII^ mice (**Fig 4E**). IFN-γ-inducible genes that were expressed in significantly lower amounts in CMs, NCMs and DCs, but not MDMs, in CCR2^ΔMHCII^ compared to CCR2^WT^ mice included chemokines (*Cxcl9*, *Cxcl10*), guanylate-binding proteins (*Gbp3*, *Gbp4*, *Gbp5*, *Gbp6*) and suppressor of cytokine signaling (SOCS) proteins (*Socs1*, *Socs3*) (**Fig 4F**). Thus, CMs, NCMs and DCs underwent a small amount of IFN-γ receptor signaling that was dependent on MHCII expression, while MDMs underwent a large amount of IFN-γ receptor signaling that was independent of MHCII expression. Importantly, these results indicated that the inability of CCR2^ΔMHCII^ mice to control *Mtb* in MDMs cannot be explained by a failure of MDMs to undergo IFN-γ receptor signaling.

### A subset of MDMs enriched for *Mtb* bacteria express glycolytic genes in response to cognate CD4^+^ T cell help

**Fig 5:**
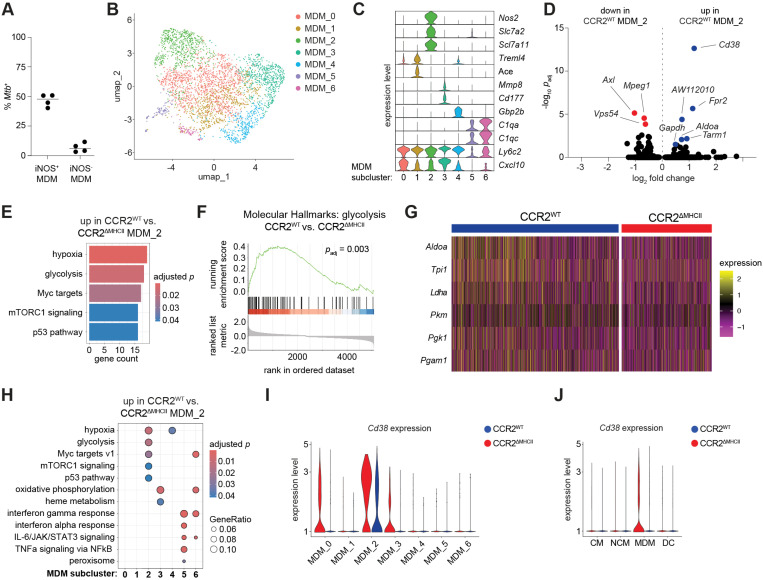
A subcluster of MDMs enriched for *Mtb* upregulate glycolysis in response to cognate interactions with CD4 ^**+**^ **T cells.** (A) Frequency of *Mtb*-mScarlet positivity among iNOS^+^ and iNOS^–^ MDMs in WT mice infected for 3 weeks with *Mtb* based on flow cytometry. (B) Subclusters of MDMs among total combined MDMs from CCR2^WT^ and CCR2^ΔMHCII^ mice. (C) Relative expression of indicated genes (right) among MDM subclusters (bottom). Full set of cluster-defining genes are presented in S4A Table. (D) Gene expression of CCR2^WT^ MDM_2 cells compared to CCR2^ΔMHCII^ MDM_2 cells. *H2-Ab1* as well as *Rpl9-ps6*, a pseudogene transcript that was absent in all cells from CCR2^ΔMHCII^ samples, were excluded from the plot. Full set of differentially-expressed genes are presented in S4B Table. (E) Biological pathways enriched within the top differentially expressed genes (log_2_ fold change > 0.5) in CCR2^WT^ MDM_2 cells compared to CCR2^ΔMHCII^ MDM_2 cells. Pathways were identified using over-representation analysis by integrating the mSigDB Molecular Hallmarks geneset collection (see Materials and Methods). Core enrichment genes are presented in S4C Table. (F) Gene set enrichment analysis (GSEA) of the genes ranked by log_2_ fold change that were preferentially expressed in the MDM_2 cells from CCR2^WT^ mice compared to the same population in CCR2^ΔMHCII^ mice, referencing the mSigDB Molecular Hallmarks “glycolysis” geneset. (G) Expression of glycolysis-related genes in MDM_2 cells from CCR2^WT^ versus CCR2^ΔMHCII^ mice that were identified by GSEA (full list of genes in S4D Table). Each column represents a cell from the indicated mice. (H) Over-representation analysis as in (E), but comparing results across MDM subclusters from the indicated mice. (I-J) *Cd38* expression levels in MDM subclusters (I) and myeloid cell clusters (J; see Fig 4A) from the indicated mice. Statistical significance was determined Wilcoxon rank sum test with Benjamini-Hochberg correction (D, E, H), hypergeometric test with Benjamini-Hochberg correction (E, H) or rank-based enrichment scoring with Benjamini-Hochberg correction (F).

It was possible that MHCII-dependent differences were limited to the subset of MDMs that contained *Mtb* bacteria. *Mtb*-infected MDMs could not be directly identified in the scRNA-seq data because this method does not detect *Mtb* mRNA molecules. When MDMs were assessed by flow cytometry, however, about 50% of iNOS^+^ but less than 10% of iNOS^–^ MDMs contained *Mtb*-mScarlet bacteria, indicating that expression of *Nos2*, the gene encoding iNOS, could be used as proxy for infection (Fig 5A). This was consistent with prior reports that most *Mtb*-infected non-AMs express *Nos2* [29]. MDMs were therefore clustered separately from the other cells in the scRNA-seq dataset to identify the *Nos2*^*+*^ subset. This analysis resolved 7 MDM sub-populations (MDM_0 through MDM_6) (Fig 5B), only one of which, MDM_2, contained cells that expressed *Nos2* as well as the arginine acquisition-related genes *Slc7a2* and *Slc7a11* [61]. Cells in MDM_0 were transcriptionally similar to those in MDM_2 but did not express the NO-promoting genes. Cells in the MDM_1 cluster had an NCM-like phenotype based on expression of *Treml4* and *Ace*, but, in contrast to NCMs, expressed *Ly6c2* at similar abundance to other MDM populations. Cells in MDM_3 expressed *Mmp8* and *Cd177*, genes associated with activated PMNs [62,63], while the MDM_4 cluster was distinguished by expression of the IFN-γ-inducible GTPase *Gbp2b* [64]. Two small clusters, MDM_5 and MDM_6, were enriched for complement transcripts (*C1q*a*, C1qc*) and expressed lower levels of *Ly6c2* and *Cxcl10* than the other MDM subclusters (Fig 5C) (full list of cluster-defining genes in (S4A Table).

*Mtb* infection-enriched MDM_2 cells were then analyzed for MHCII-dependent pathways. MDM_2 cluster cells from CCR2^WT^ mice expressed more *Cd38*, *Aldoa*, *Gapdh, Fpr2*, *Tarm1*, and *Aw112010* and less *Axl*, *Mpeg1*, and *Vps54* than MDM_2 cells from CCR2^ΔMHCII^ mice (Fig 5D) (S4B Table). The increase in *Aldoa* (aldolase A) and *Gapdh* (glyceraldehyde-3-phosphate dehydrogenase) expression indicated that glycolytic metabolism was upregulated in an MHCII-dependent manner. Upregulation of *Cd38,* a prominent feature of *Mtb*-infected lung cells [24,65], was similarly notable because CD38 was recently found to promote glycolysis by stabilizing HIF-1α [65]. To assess MHCII-dependent gene expression in an unbiased fashion, biological pathway analysis was performed on the top differentially expressed genes between MDM_2 cells from CCR2^WT^ and CCR2^ΔMHCII^ mice. Genes related to hypoxia and glycolysis were preferentially expressed by the MDM_2 cells of CCR2^WT^ mice along with mTORC1, c-Myc and p53, regulators that promote glycolytic metabolism [66,67] (Fig 5E, genes in core enrichments listed in S4C Table).

Upregulation of glycolysis-related genes was notable because macrophage glycolysis is strongly associated with *Mtb* control in other studies [23,30,68] and is a defining feature of *Mtb*-infected MDMs in WT mice [28,29]. GSEA was performed as an additional approach to test if MDM expression of MHCII molecules, and presumably receipt of signals from cognate CD4^+^ T cells, led to upregulation of glycolytic genes in MDM_2 cells. Indeed, genes associated with glycolysis were significantly enriched among the upregulated genes, ranked by log_2_ fold change, in CCR2^WT^ cells compared to CCR2^ΔMHCII^ cells in this cluster (Fig 5F; genes in core enrichment listed in S4D Table). Among the core enrichment genes, the glycolytic enzymes aldolase (*Aldoa*), triosephosphate isomerase (*Tpi1*), lactate dehydrogenase (*Ldha*), pyruvate kinase *(Pkm*), phosphoglycerate kinase *(Pgk1*), and phosphoglycerate mutase (*Pgam1*) were upregulated in many MDM_2 cells from CCR2^WT^ mice compared to CCR2^ΔMHCII^ mice (Fig 5G).

A final set of analyses were performed to test if MHCII-dependent upregulation of glycolytic genes was a unique feature of MDM_2 cells compared to the other MDM subclusters. Comparative over-representation analysis showed that MHCII-dependent upregulation of genes associated with hypoxia, glycolysis, Myc, mTORC1 and p53, while observed in MDM_2 cells, was not consistently observed for the other MDM subclusters. Instead, MDM_3 cells from MHCII^ΔCCR2^ mice were defective in the expression of genes related to oxidative phosphorylation and heme metabolism, while the small subclusters MDM_5 and MDM_6 were defective in the expression of peroxisome and inflammatory cytokine-induced genes in MHCII^ΔCCR2^ mice (Fig 5H). Similarly, while *Cd38* was upregulated by MDM_2 cells in an MHCII-dependent manner, its expression was minimal in the other MDM subclusters regardless of MHCII status (Fig 5I) and absent from CMs, NCMs or DCs (Fig 5J). Overall, the results showed that the subset of MDMs most highly enriched for *Mtb* bacteria upregulate glycolytic genes in an MHCII-dependent manner. The loss of *Mtb* protection observed in MHCII^ΔCCR2^ mice may therefore be explained by a failure of infected MDMs to increase glycolytic metabolism.

### CD4^+^ T cells promote MDM expression of CD38, which contributes to *Mtb* control

**Fig 6:**
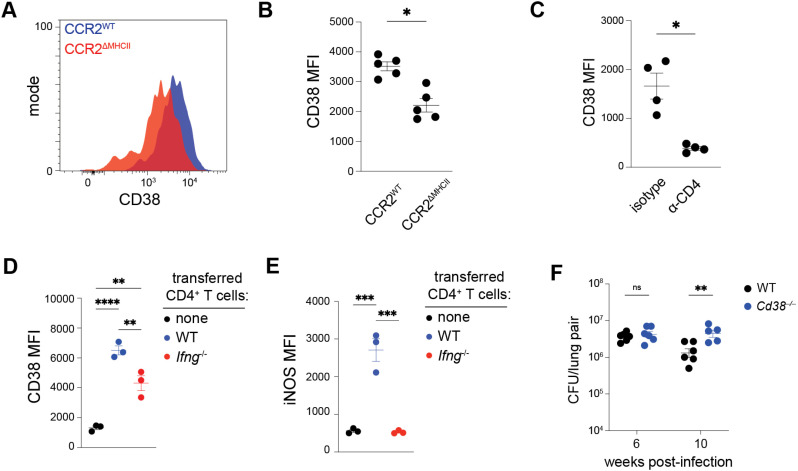
CD4 ^**+**^ **T cells promote MDM expression of CD38, which contributes to *Mtb* control.** (A-B) Mean fluorescence intensity (MFI) of CD38 antibody staining of infected MDMs in indicated mice. (C) CD38 MFI of infected MDMs in WT mice treated with isotype or CD4 + T cell-depleting (α-CD4) antibodies. (D) CD38 MFI and (E) iNOS MFI of infected MDMs in T cell-deficient (*Tcra*^–/–^) mice receiving adoptively transferred CD4^+^ T cells from naïve WT or IFN-γ-deficient (*Ifng*^–/–^) donors, or receiving no transferred cells, prior to *Mtb* infection. (F) *Mtb* colony-forming units (CFUs) in the lungs of WT or CD38-deficient mice. Data were collected at 3 weeks (A-C) or 4 weeks (D-E) post-infection. Symbols represent n = 5 (B), n = 4 (C), n = 3 (D-E), and n ≥ 5 (E) biological samples from 2 independent experiments per group with the group the mean ± SEM. Statistical significance was determined by unpaired t test with Welch’s correction (B-D) and one- or two-way ANOVA with Šidák correction (D-F). *p < 0.05, **p < 0.01, ***p < 0.001, ****p < 0.0001.

The most significantly downregulated gene in MHCII-deficient MDM_2 cells was *Cd38* (**Fig 5D**), an enzyme that can promote glycolysis in part by stabilizing HIF-1α [65,69] and was recently correlated with protection against *Mtb* [70]. CD38 is also expressed on the cell surface and is readily detected by flow cytometry. This feature was used to further validate the transcriptional evidence that cognate CD4 + T cell help to infected MDMs increases their glycolytic phenotype. Infected MDMs from CCR2^ΔMHCII^ mice had significantly weaker CD38 staining compared to CCR2^WT^ mice at 3 weeks post-infection (**Fig 6A and 6B**),  mirroring the defect in *Cd38* transcription observed in MDM_2 cells from CCR2^ΔMHCII^ mice. This effect was dependent on CD4^+^ T cells because infected MDMs from CD4^+^ T cell-depleted mice had lower CD38 staining than infected MDMs from intact mice (**Fig 6C**). Thus, optimal expression of CD38 by infected MDMs requires both CD4^+^ T cells and MHCII on monocyte-derived cells.

While *Cd38* expression by *Mtb*-infected macrophages was previously associated with IFN-γ stimulation [24], our earlier transcriptional analysis suggested that IFN-γ signaling is not sufficient to explain MHCII-dependent upregulation of *Cd38* and other glycolysis-related genes in *Mtb*-infected MDMs. Supporting this notion, mice with either WT or IFN-γ-deficient CD4^+^ T cells had significantly higher CD38 levels on infected MDMs compared to T cell-deficient mice, although there was a modest decrease in CD38 levels in the absence of CD4^+^ T cell-derived IFN-γ (**Fig 6D**). By contrast, expression of the IFN-γ-inducible protein iNOS by infected MDMs was only observed in mice with WT CD4^+^ T cells (**Fig 6E**). Thus, CD4^+^ T cells promoted CD38 expression by infected MDMs independently of IFN-γ and this activity was likely explained by cognate MHCII-mediated interactions.

Finally, we determined whether CD38 contributed to *Mtb* control. WT and CD38-deficient (*Cd38*^–/–^) mice were infected with *Mtb* and the total lung bacterial burden was measured at 6 and 10 weeks post-infection. At 6 weeks post-infection, there was no difference in lung *Mtb* burden between WT and CD38-deficient mice, but by 10 weeks post-infection there were approximately 3-fold more bacteria in CD38-deficient mice compared to WT mice (**Fig 6F**). This result contrasted with observations of CCR2^ΔMHCII^ mice, which had nearly 100-fold more *Mtb* bacteria in the lungs compared to CCR2^WT^ control mice as early as 6 weeks post-infection (**Fig 3C**). Thus, while CD38 activity contributes to *Mtb* control, CD38 alone cannot fully account for MHCII-dependent attenuation of *Mtb* growth in MDMs.

## Discussion

A goal of this study was to determine how CD4^+^ cells control *Mtb* infections in lung myeloid cells. It was important in this regard to identify the myeloid cells that are infected with *Mtb* during CD4^+^ T cell-mediated control and described in earlier work as CD11c^int^ MHCII^int^ IM cells expressing a mix of macrophage and DC surface markers [22–25,27]. Using an intravascular labeling technique and a flow cytometry panel including CX_3_CR1 and Ly-6C antibodies, we ruled out CM and NCM blood contaminants and cells of DC origin and showed that the major *Mtb*-infected myeloid population in the lung interstitium several weeks after infection are MDMs. It was notable that MDMs were preferentially infected compared to DCs, despite both of these cells being present in similar numbers. This result indicated that MDMs are uniquely poised to phagocytose *Mtb* bacteria, potentially by expressing surface receptors that recognize components of the *Mtb* envelope [71]. While AMs are the first cells to phagocytose inhaled *Mtb* [22], our results are consistent with prior reports that non-AMs and PMNs become the dominant locations of *Mtb* by 3 weeks post-infection following a standard low-dose (~100 CFU) aerosol challenge [22,24,72]. The non-AMs were confirmed by our study to be MDMs and were thus the focus of our analysis. While we found that CD4^+^ T cell recognition of peptide:MHCII complexes on monocyte-derived cells is a prerequisite for *Mtb* control, we cannot exclude the possibility that CD4^+^ T cell signaling to AMs contributes to immunity as well.

Our MDM recruitment studies revealed an unrecognized function of CD4^+^ T cell-derived IFN-γ, which both recruited CMs from blood into the lung interstitium and promoted differentiation into MDMs. These observations clarify prior findings that mice deficient in CD4^+^ T cells or IFN-γ had reduced numbers of myeloid cells in the lungs during *Mtb* infection [19,24,72]. Our results are also consistent with prior reports that CD4^+^ memory T cells elicited by BCG immunization promote accelerated recruitment of MDMs to the lungs upon *Mtb* infection [26] and further demonstrate that IFN-γ secreted by the CD4^+^ T cells explains this effect. It was notable, however, that CMs did not require IFN-γ receptor expression to extravasate and differentiate into lung-resident MDMs. This result indicates that IFN-γ promotes CM extravasation by an indirect mechanism, perhaps by signaling directly to endothelial cells. In support of this notion, a previous study found that IFN-γ receptor deficiency in non-hematopoietic cells resulted in fewer CD11b^+^ CD11c^+^ cells in the lungs [73]. In a murine model of colitis, direct IFN-γ signaling to endothelial cells was required for vascular permeabilization and infiltration of leukocytes into inflamed tissue [74]. IFN-γ also induces the expression of chemokines and endothelial surface proteins that promote extravasation [57,75]. Thus, IFN-γ secreted by *Mtb-*specific CD4^+^ T cells may increase chemokine expression or activate lung endothelial cells to promote CM migration from the blood into the lung tissue.

The lung antigen-presenting cells that stimulated *Mtb-*specific CD4^+^ T cells to secrete IFN-γ and recruit monocytes were not MDMs as evidenced by the observation that mice lacking MHCII molecules on all cells with a history of CCR2 expression had normal amounts of IFN-γ in the lungs. Thus, cells other than CMs, NCMs, MDMs or CCR2-expressing DCs present antigens to *Mtb*-specific CD4^+^ T cells within the lungs to maintain CD4^+^ T cell survival and IFN-γ secretion. It is possible that AMs are an important source of *Mtb* antigen presentation for these processes because AMs are the first cells to become infected with inhaled *Mtb* and migrate into the lung interstitium shortly before the CD4^+^ T cell response is initiated [22,25]. Alternatively, a rare antigen presenting cell population that was not identified by this study could be required for stimulating lung-resident CD4^+^ T cells. Lastly, cytokines such as IL-12 or IL-18 produced within *Mtb*-infected lungs could maintain CD4^+^ T cell survival and IFN-γ secretion independently of TCR signaling, as has been reported elsewhere [76].

The recruitment of infectable MDMs by CD4^+^ T cells may be advantageous to the host by luring *Mtb* bacteria into host cells that are capable of presenting MHCII-bound *Mtb* peptides to CD4^+^ T cells and receiving microbicidal signals. Importantly, this signal could not be accounted for by IFN-γ alone. MDMs underwent a high degree of IFN-γ receptor signaling regardless of their ability to present MHCII-bound *Mtb* peptides to cognate CD4^+^ T cells but required cognate interactions to fully upregulate glycolytic gene expression and control infection. IFN-γ receptor signaling may however still contribute an MHCII-independent component of protection. A prior study of mixed bone marrow chimeric mice demonstrated that IFN-γ receptor-deficient leukocytes contained more *Mtb* bacteria than IFN-γ receptor-proficient leukocytes within the lungs of the same animal [77]. Moreover, *in vitro* studies have demonstrated that IFN-γ can promote glycolysis in *Mtb*-infected BMDMs in part by stabilizing HIF-1α [30,78]. Lastly, IFN-γ plays a separate role in protecting hosts from neutrophil-induced lung damage during Mtb infection [15,77]. While the contribution of IFN-γ to the metabolic programming of lung MDMs requires further investigation, the results of our study indicate that the MHCII-dependent component of *Mtb* control by MDMs *in vivo* occurs in an IFN-γ-independent manner.

While it is clear that IFN-γ from one or more cellular sources is essential for *Mtb* protection in humans and mice [3,9,13,79], it remains debated whether IFN-γ secretion by *Mtb*-specific CD4^+^ T cells is necessary for their antimicrobial activity. We found that adoptive transfer of WT or IFN-γ-deficient CD4^+^ T cells into *Tcra*^–/–^ hosts produced a similar degree of *Mtb* control in the lungs at 4 weeks post-infection. Similar results were observed by other groups when using T- and B cell-deficient *Rag1*^–/–^ mice as adoptive transfer recipients, although IFN-γ-dependent control by transferred CD4^+^ T cells became significant later at time points [14,19,80]. By contrast, IFN-γ secretion by CD4^+^ T cells was shown to be critical for protective activity as early as 4 weeks post-infection when transferred into recipient mice lacking both αβ and γδ T cells (*Trcb*^–/–^*Tcr*δ^–/–^) [80]. These discrepant results may be explained by differences in the total abundance of IFN-γ between *Tcra*^–/–^, *Trcb*^–/–^*Tcr*δ^–/–^, and *Rag1*^–/–^ mice or differences in the number of adoptively transferred CD4^+^ T cells. Thus, the non-physiological nature of adoptive transfer studies may not accurately capture CD4^+^ T cell function in an unmanipulated host, and non-T cell sources of IFN-γ may be sufficient to achieve protection in many contexts.

The metabolic shift of macrophages towards glycolysis is required for control of *Mtb* and other intracellular bacterial pathogens [30,81–83]. Our study suggests that the high degree of glycolytic gene expression observed in *Mtb*-infected MDMs [28,29] may be partly attributed to MHCII-mediated signaling from cognate CD4^+^ T cells. Although we were not able to directly quantify glycolytic flux in infected lung MDMs due to the low number of cells that could be isolated from mice, we detected increased activity of the pro-glycolytic regulators mTORC1, c-Myc and p53 [66,67], including the expression of enzymes directly involved in glycolysis, in the MDMs that received cognate CD4^+^ T cell help. Importantly, prior studies suggest that macrophage glycolysis supports human immunity to tuberculosis. Glycolytic macrophages are observed in pre-necrotic *Mtb* lesions from human lungs and nicotinamide, which supports glycolysis through its conversion to NAD cofactor, is an effective antitubercular drug in humans [78,84]. The mechanisms by which macrophage glycolysis supports antimicrobial immunity are poorly understood but may include limiting metabolites that support bacterial growth, producing microbicidal metabolites, or meeting demands for additional biochemical processes in the macrophage. Glycolysis supports the citric acid cycle and pentose phosphate pathways, which are important for generating antimycobacterial molecules such as itaconate and reactive oxygen species (ROS) [85,86]. Moreover, the preference for glycolysis over fatty acid oxidation (FAO) may be important for *Mtb* control because FAO mitigates mitochondrial ROS production during *Mtb* infection [87]. Thus, the induction of MDM glycolysis via cognate CD4^+^ T cell help may restrict *Mtb* growth through multiple mechanisms.

CD4^+^ T cells from *Mtb-*infected mice were shown by a previous study to suppress *Mtb* growth within bone marrow-derived macrophages in a HIF-1α-dependent manner and induce hypoxia- and glycolysis-related genes [88]. The present study supports these earlier observations and further establishes that induction of glycolysis in infected MDMs is triggered by antigen presentation to CD4^+^ T cells *in vivo* and may be promoted in part by CD38. Interactions between ligands that are acutely induced on CD4^+^ T cells by TCR recognition of peptide:MHCII complexes and receptors on MDMs that display such complexes, such as CD40 or other tumor necrosis factor-family receptors, are good candidates for the transducers of the signals that induce glycolysis in MDMs.

## Materials and methods

### Ethics statement

All experiments were performed according to the guidelines and approval of the University of Minnesota Institutional Animal Care and Use Committee, protocol number 2404-42019A and the University of Minnesota Institutional Biosafety Committee, protocol number 2501-42720H.

### Animals

Mice were housed under specific pathogen-free conditions in accordance with University of Minnesota Institutional Animal Care and Use Committee guidelines. C57BL/6J and B6.SJL-Ptprca Pepcb/BoyJ (CD45.1) mice were bred in-house. The following mouse lines were purchased from Jackson Laboratories and bred in-house: B6.129S2-Tcratm1Mom/J (*Tcra*^*-/-*^), C57BL/6J-Ms4a3em2(cre)Fgnx/J (*Ms4a3*^Cre^), B6.Cg-Gt(ROSA)26Sortm9(CAG-tdTomato)Hze/J (tdTomato^LSL^), B6.129S7-Ifngtm1Ts/J (*IFNg*^-*/-*^), B6.129S7-Ifngr1tm1Agt/J (*IFNgR1*^*-/-*^), C57BL/6-Ccr2em1(icre/ERT2)Peng/J (*Ccr2-CreERT2-GFP*), B6.129X1-H2-Ab1b-tm1Koni/J (*H2-Ab1*^*fl/fl*^), B6.129P2-Cd38tm1Lnd/J (*Cd38*^–/–^). *Ms4a3*^Cre^ and tdTomato^LSL^ mice were bred for one generation to produce mice heterozygous for both alleles. CCR2^WT^ and CCR2^ΔMHCII^ mice were generated by crossing *Ccr2-CreERT2-GFP* and *H2-Ab1*^*fl/fl*^ for several generations to produce litters of *H2-Ab1*^*fl/fl*^ mice that were either heterozygous or null for the *Ccr2-CreERT2-GFP* allele. Littermates were used for experiments. Mice used for experiments were 4–8 weeks old with the exception of bone marrow chimeric mice, which were 4–8 weeks old at the time of irradiation and 14–18 weeks old at the time of infection. CCR2^WT^ and CCR2^ΔMHCII^ mice were fed with chow containing tamoxifen (500 mg/kg diet formulation, Inotiv Teklad) starting on the day of *Mtb* challenge. Mice were age-matched for experiments and approximately equal proportions of male and female mice were used.

### Bacterial strains and plasmids.

The *Mycobacterium tuberculosis* strain H37Rv was transformed with a chromosomally integrating plasmid expressing a variant of mScarlet fluorescent protein [89] to generate strain *Mtb*-mScarlet. This plasmid is selectable by hygromycin resistance, using the mycobacterial optimized promoter (MOP) to drive mScarlet expression and a Giles phage integration module [90].

To make fluorescent H37Rv expressing 2W peptide, an open reading frame encoding H37Rv EsxA (Rv3875) with a C-terminal 4 x glycine linker, followed by 2W peptide [46], followed by a FLAG peptide was inserted into pMN402 [91]. pMN402 contains the promoter region of H37Rv *hsp60* (Rv0440) upstream of the insertion site. The promoter and coding sequence were subcloned into pTT1B, which encodes gentamycin resistance and chromosomally integrates into the *Mtb* L5 integration locus [92]. The resulting plasmid (pTT1B-EsxA-2W) was transformed into *Mtb*-mScarlet by electroporation and transformants were selected on hygromycin- and gentamycin-containing agar plates.

### Mouse infections

Mice were infected with a low dose (~100 CFU/lung pair) of *Mtb* via aerosol exposure. *Mtb* strains were grown from frozen stocks in complete 7H9 broth (Remel Middlebrook 7H9 broth with 0.2% glycerol, 0.5% BSA, 0.2% dextrose, 0.085% sodium chloride and 0.05% Tween-80). For preparing the bacterial inoculum, *Mtb* was grown to an optical density at 600 nm (OD600) of 0.4-0.6, washed in PBS-T (PBS containing 0.5% Tween-80) and diluted to an OD600 of 0.005. The inoculum was nebulized for aerosol delivery using the Glas-Col inhalation exposure system. The infectious dose was monitored by plating total lung homogenate the day after aerosol exposure. For CD4^+^ T cell depletion, α-CD4 or isotype control antibodies were injected intraperitoneally weekly starting on the day of infection.

### Processing of infected lungs for flow cytometry and cell sorting

Mice were euthanized using carbon dioxide inhalation in accordance with University of Minnesota Institutional Animal Care and Use Committee guidelines. Mouse lungs were placed in IMDM complete media (IMDM supplemented with GlutaMAX, pyruvate, non-essential amino acids, 10% fetal bovine serum and 200 µM beta-mercaptoethanol) (Gibco) and homogenized using a GentleMACS tissue dissociator (Miltenyi). Homogenates were strained through a 70 µm filter to generate single cell suspensions.

### Cell staining and counting for flow cytometry and cell sorting

Single cell suspensions were split into multiple samples for T cell and myeloid cell staining. For T cell staining, cells were stained with fluorophore-conjugated antibodies (1:50 dilution), peptide:MHCII tetramers (1:100 dilution), and viability dye (1:500 dilution) for 20 minutes at room temperature. For myeloid cell staining, cells were stained with fluorophore-conjugated antibodies (1:50 dilution) and viability dye (1:500 dilution) for 20 minutes at room temperature. For intracellular staining with iNOS antibody, cells were treated with fixation/permeabilization solution (BD), washed with permeabilization wash buffer (Tonbo), and stained in permeabilization wash buffer for 20 minutes at room temperature. For all samples subject to flow cytometry analysis, cells were fixed overnight in 5% formalin at 4ºC for sterilization. For i.v. labeling experiments, mice were injected retro-orbitally with 2.5 µg of fluorophore-conjugated CD45 antibody 3 minutes prior to euthanasia. To obtain cell counts, AccuCheck Counting Beads (ThermoFisher Scientific) were added to the fixed cell samples immediately before analysis on the flow cytometer.

### Monocyte and CD4^+^ T cell purification and adoptive transfer

Monocytes purified from donor bone marrow using the Stemcell Mouse Monocyte Isolation Kit were retro-orbitally injected into *Mtb-*infected recipient mice, 2–4 x 10^6^ monocytes per recipient. For purification and adoptive transfer of CD4^+^ T cells, lymph nodes and spleens of infection-naïve donor mice were pooled, homogenized using a GentleMACS tissue dissociator and strained through a 70 µm filter to generate a single-cell suspension. CD4^+^ T cells were then purified by negative selection and magnetic separation as follows. Cells in IMDM complete media were incubated with biotinylated antibodies against CD11b, CD11c, CD16/32, B220, CD8a, Ter119, NK1.1 and F4/80 (1:100 dilution) for 15 minutes at room temperature. Stained cells were mixed with streptavidin-conjugated magnetic beads (Stemcell) for 3 minutes, after which a larger volume of complete media was added and the samples were applied to a magnet (Stemcell) for 3 minutes. CD4^+^ T cells were collected by recovering the unbound fraction. CD4^+^ T cells purified from donor mice were resuspended in PBS and retro-orbitally injected into infection-naïve recipient mice, approximately 20 million CD4^+^ T cells per recipient. The CD4^+^ T cell-recipient mice were rested for 7 days before challenge with *Mtb*.

### Enumeration of bacterial colony-forming units

Mouse lungs were homogenized as described above. The homogenate was mixed 1:1 with PBS-T without prior straining. Serial dilutions were prepared in PBS-T and plated onto Remel 7H11 agar containing 0.5% glycerol and supplemented with 10% BD BBL Middlebrook OADC Enrichment (containing catalase and oleic acid).

### Cytokine quantification

*Mtb*-infected mouse lungs were homogenized as described above. A fraction of the homogenate was centrifuged at 10,000 x g for 5 minutes at 4ºC. The supernatant fraction was transferred to a Spin-X 22 µm filter centrifuge tube (Corning) and centrifuged for 30 minutes at 4ºC. The filtrate was collected and stored at -20ºC until assayed. Cytokines were measured in the samples using the LEGENDplex Mouse Inflammation Panel ELISA kit (BioLegend). Concentrations were obtained using standards provided in the kits according to the manufacturer guidelines.

### Generation of mixed bone marrow chimeric mice

Mice were irradiated with 500 rad in an X-ray irradiator, rested for 24 hours, and irradiated a second time with 500 rad. Bone marrow was collected from donor mice in DMEM media (Gibco) containing 10% fetal bovine serum and counted using a hemocytometer. 2.5 x 10^5^ bone marrow cells from each donor were pooled and transferred into irradiated recipient mice via retro-orbital injection. Mice were rested for approximately 10 weeks prior to infection to allow reconstitution of their hematopoietic systems.

### Single cell RNA sequencing

Single cell suspensions were generated from the lungs of 2 CCR2^WT^ mice (1 male and 1 female) and 2 CCR2^ΔMHCII^ mice (1 male and 1 female) and kept at 4ºC in IMDM complete media. Cells were stained with antibodies against Thy1.2, B220, CD19, Ly-6G, Siglec-F, CD11b, and NK1.1 (1:25 dilution) and viability dye (1:500 dilution) for 30 minutes. Cells staining positively for CD11b and negatively for the remaining markers were sorted using a Sony MA-900 cell sorter. Cells were sorted into IMDM complete media with 40% FBS. Approximately 30,000 cells per sample were recovered from the sorter. Sorted cell samples were processed to obtain RNA in separate wells of the same microfluidics chip using the Chromium Next GEM Single Cell 3’ Gene Expression kit and Chromium X controller (10X Genomics). cDNAs were amplified and libraries barcoded by sample ID were generated according to the manufacturer instructions. Libraries were sequenced by the DNA Services lab at the University of Illinois at Urbana-Champaign. For sequencing, the libraries were pooled, quantitated by qPCR and sequenced on 2 10B lanes with 28 x 150 nucleotide reads on a NovaSeq X Plus with V1.0 sequencing kits (Illumina). The samples each yielded 350–450 million paired reads.

### Analysis of RNA sequencing data

Analysis of single cell RNA sequencing data was performed in R. Using the Seurat package [93], mitochondrial gene features were identified and cells with a mitochondrial feature content of greater than 5% were counted as non-viable and removed from the analysis. Cells with outlying feature counts (under 500 or over 4,000) were also removed. The feature counts for each cell were normalized and scaled using default Seurat parameters as follows. For normalization, the count of each feature was divided by total counts for that cell and multiplied by 10,000. For scaling, features were centered to have a mean of 0 and scaled by the standard deviation of that feature. Scaled data was used to identify variable features, run principal component analysis, and perform differential gene expression analysis. SingleR [94] was used to identify and remove contaminating lymphocytes, NK cells and PMNs by integrating reference data from ImmGen [95]. The clusterProfiler package [96] was used to perform over-representation analysis and GSEA. For GSEA, differentially expressed genes were ranked in decreasing order of log_2_ fold change. The IFN-γ response module (Fig 4E) consisted of genes within the mSigDB “interferon gamma response” geneset [56] that were enriched in CMs from CCR2^WT^ versus CCR2^ΔMHCII^ mice based on GSEA (genes are listed in S3D Table). For differential gene expression, over-representation and GSEA analyses, statistical significance was determined Wilcoxon rank sum test with Benjamini-Hochberg correction.

### Data packages

The single-cell RNA sequencing data has been deposited in NCBI’s Gene Expression Omnibus [97] and is accessible through GEO Series accession number GSE287753. All other raw data used for this publication has been deposited in a Dryad public repository [98].

## Supporting information

S1 Fig Additional data related to Fig 1. (PDF)

S2 Fig Additional data related to Fig 2. (PDF)

S3 Fig Additional data related to Fig 3. (PDF)

S4 Fig Additional data related to Fig 4. (PDF)

S1 Table Cluster-defining genes for CMs, NCMs, MDMs and DCs, related to Figs 4A and S4A. (XLSX)

S2 Table Differentially expressed genes between CCR2^WT^ and CCR2^ΔMHCII^ mice for CMs (A), NCMs (B), MDMs (C) and DCs (D). Related to Fig 4C.
(XLSX)

S3 Table Over-representation analysis of differentially expressed genes between CCR2^WT^ and CCR2^ΔMHCII^ mice for CMs (A), NCMs (B), and DCs (C) and GSEA core enrichment genes related to interferon gamma response for CMs (D). Related to Figs 4C-E.
(XLSX)

S4 Table (A) Cluster-defining genes for MDM subclusters MDM_0 through MDM_6. (B-D) Differentially expressed genes (B), over-representation analysis (C) and GSEA core enrichment genes related to glycolysis (D) between MDM_2 cells from CCR2^WT^ and CCR2^ΔMHCII^ mice. Related to Fig 5.
(XLSX)
