# Supplementary material for: Antigen-specific CD4+ T cells promote monocyte recruitment and differentiation into glycolytic lung macrophages to control Mycobacterium tuberculosis
Source: PLoS Pathog. 2025 Jun 9;21(6):e1013208. doi: 10.1371/journal.ppat.1013208 (PMC12193047; doi:10.1371/journal.ppat.1013208)
Supplement: S1 Fig — (PDF) [file ppat.1013208.s001.pdf]

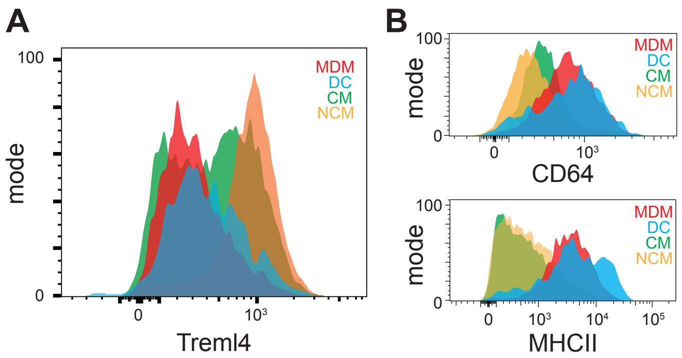

Figure S1. Additional data related to Figure 1. (A) Treml4 antibody staining of indicated cells in the lungs of infected WT mice. (B) CD64 and MHCII antibody staining of indicated cells in the lungs of infected WT mice. Data was collected at 3 weeks post-infection.
