# Supplementary material for: Antigen-specific CD4+ T cells promote monocyte recruitment and differentiation into glycolytic lung macrophages to control Mycobacterium tuberculosis
Source: PLoS Pathog. 2025 Jun 9;21(6):e1013208. doi: 10.1371/journal.ppat.1013208 (PMC12193047; doi:10.1371/journal.ppat.1013208)
Supplement: S2 Fig — (PDF) [file ppat.1013208.s002.pdf]

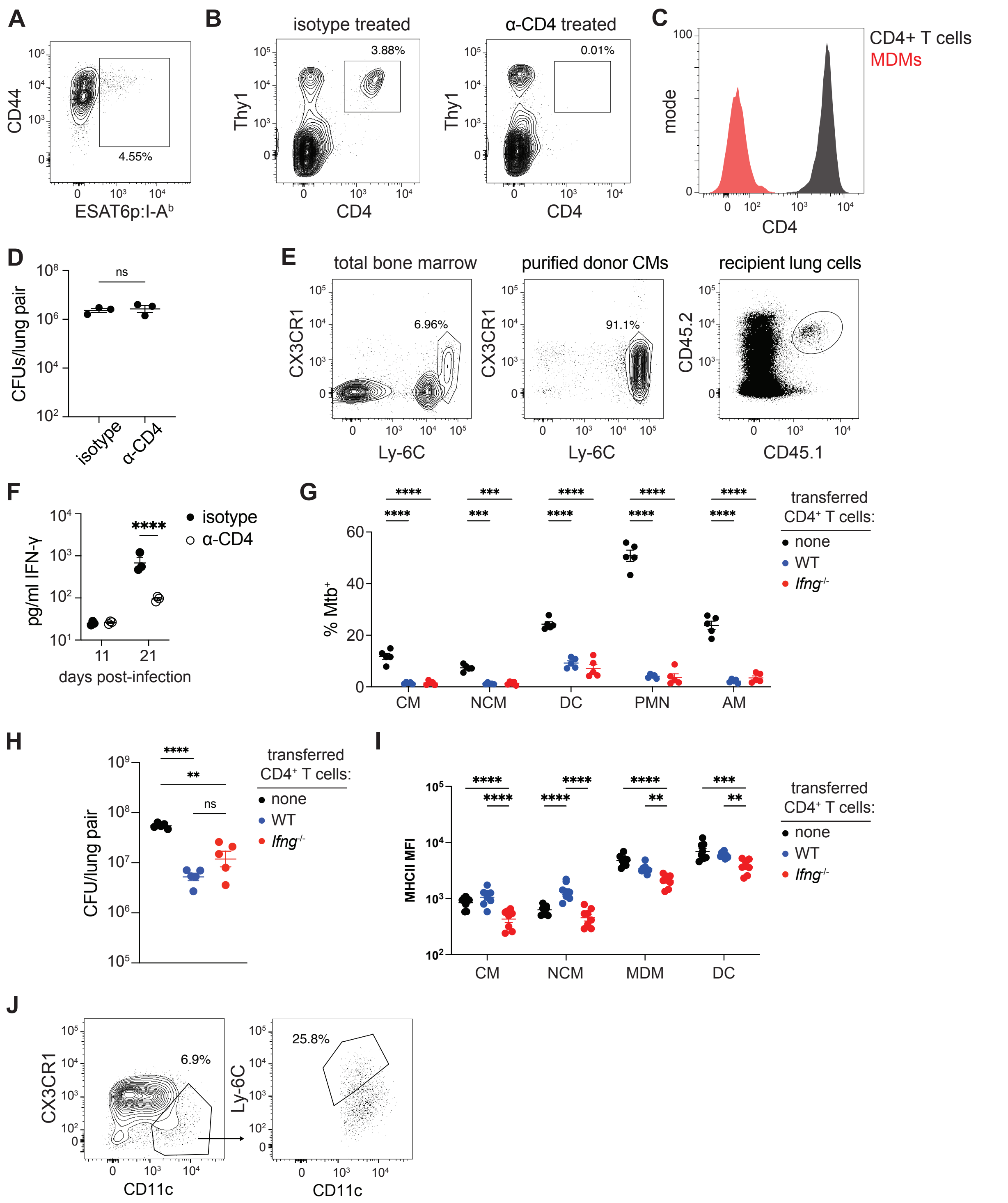

Figure S2. Additional data related to Figure 2. (A) Staining of lung CD4$^+$ T cells with ESAT6p:I-A^b tetramer. (B) Lung CD4$^+$ T cell abundance in mice treated with α-CD4 or isotype control antibody. (C) Staining of CD4$^+$ T cells and MDMs with a fluorescent CD4 antibody of the same clone (GK1.5) used for CD4$^+$ T cell depletion. (D) Number of *Mtb* CFUs at 3 weeks post-infection in the lungs of mice treated with α-CD4 or isotype control antibody. (E) Purification of CMs from the bone marrow of donor CD45.1$^+$ mice (left, middle) and identification of donor CMs in the

lungs of *Mtb*-infected recipient mice (right). (F) Total IFN-γ abundance in lung homogenates from WT mice treated with $CD4^+$ T cell-depleting or isotype control antibodies. Data is representative of ≥ 2 independent experiments and the group mean ± SEM is shown. (G) Frequency of *Mtb*-$mScarlet^+$ cells among indicated lung myeloid cell populations, (H) number of *Mtb* colony-forming units (CFUs) in lung homogenates, and (I) mean fluorescence intensity (MFI) of MHCII fluorescent antibody staining among indicated lung myeloid cell populations from T cell-deficient $Tcra^{-/-}$ mice containing adoptively transferred $CD4^+$ T cells from infection-naive WT or $Ifng^{-/-}$ donor mice or containing no transferred $CD4^+$ T cells. Data for G-I represents n = 5 biological samples per group from 2 independent experiments with the group the mean ± SEM. Statistical significance was determined by unpaired t test with Welch's correction (D), one-way ANOVA with Tukey correction (H), or two-way ANOVA with Šidák correction (F, G, I). $^{**}p < 0.01$, $^{****}p < 0.0001$. (J) Identification of MDMs in the lungs of mice containing 50% WT, 50% $Ifngr1^{-/-}$ bone marrow.
