# Supplementary material for: Antigen-specific CD4+ T cells promote monocyte recruitment and differentiation into glycolytic lung macrophages to control Mycobacterium tuberculosis
Source: PLoS Pathog. 2025 Jun 9;21(6):e1013208. doi: 10.1371/journal.ppat.1013208 (PMC12193047; doi:10.1371/journal.ppat.1013208)
Supplement: S3 Fig — (PDF) [file ppat.1013208.s003.pdf]

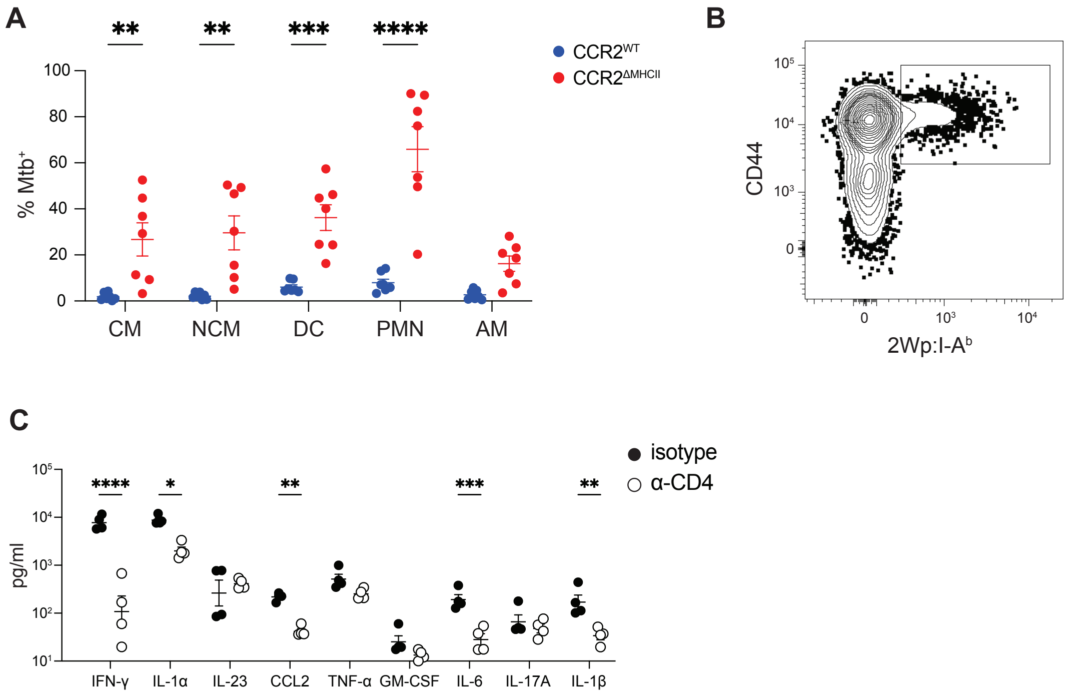

Figure S3. Additional data related to figure 3. (A) Frequency of infected cells among indicated myeloid populations at 6 weeks post-infection. (B) Staining of lung CD4$^+$ T cells with 2Wp:I-A^b tetramer. (C) Abundance of indicated cytokines at 3 weeks post-infection in the lung homogenates from WT mice treated with isotype or CD4$^+$ T cell-depleting antibody. For A and C, symbols represent n = 7 (A) and n = 4 (C) biological samples from 2 independent experiments with the group the mean ± SEM. Statistical significance was determined by two-way ANOVA with Šidák correction. *$p < 0.05$, **$p < 0.01$, ***$p < 0.001$.
