# Supplementary material for: Antigen-specific CD4+ T cells promote monocyte recruitment and differentiation into glycolytic lung macrophages to control Mycobacterium tuberculosis
Source: PLoS Pathog. 2025 Jun 9;21(6):e1013208. doi: 10.1371/journal.ppat.1013208 (PMC12193047; doi:10.1371/journal.ppat.1013208)
Supplement: S4 Fig — (PDF) [file ppat.1013208.s004.pdf]

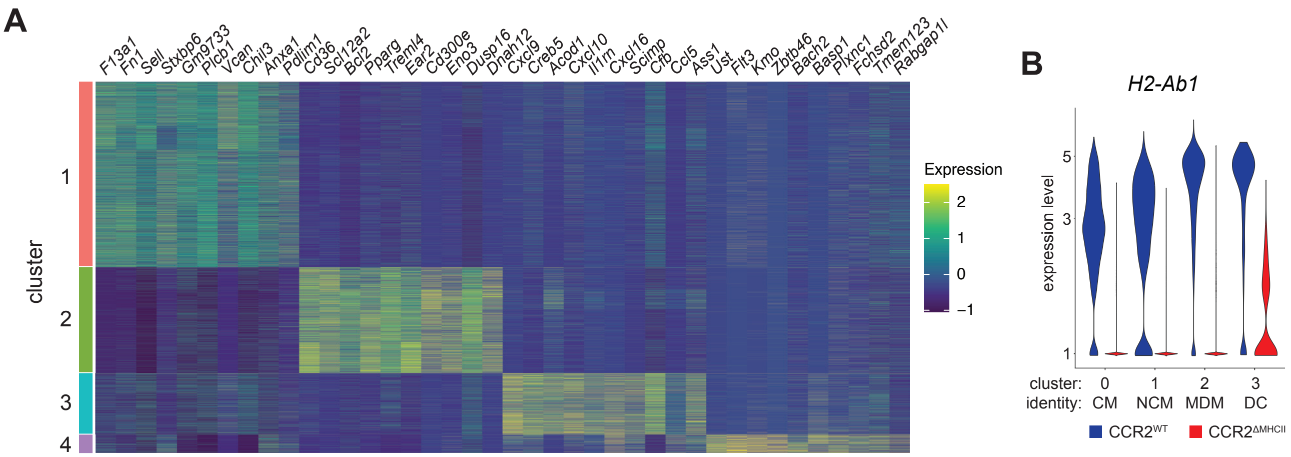

Figure S4. Additional data related to Figure 4. (A) Expression levels of top 10 cluster-defining genes among the indicated clusters, in reference to 4A. Each row represents an individual cell. (B) Aggregate expression of *H2-Ab1* (encoding MHCII) among the indicated cell clusters from CCR2^{WT} and CCR2$^{\Delta MHCII}$.
